# Wise Workers

**Published:** 1863-03

**Authors:** 


### WISE WORKERS.

About half a dozen men, who are gentlemen as well as Christians, have accomplished more within a few months, for the better observance of the Sabbath day in New York city, than has been done in twice the number of years by the whole legislative power of the City and State put together. By a steady, earnest, courteous manner of procedure, they have, in a measure, put down the Sunday press, Sunday news crying, and Sunday grog selling. Not long ago, the editors of newspapers published on the Sabbath day, claimed it as a right to issue their papers when they pleased, and branded as tyrannous and puritanical, the effort to prevent boys from offering them for sale, at the top of their voices, in the neighborhood of churches, and to all who were going and returning. But they have been compelled, at least practically, to withdraw their claims; and some of the more respectable have made Saturday their time of issue. Sabbath after Sabbath passes, and not a cry is heard from newspaper venders, where once innumerable screeches disturbed the sleepers on the early Sabbath morning, and, on the 20th of June, the morning papers announced the gratifying fact that, "The corner groceries and the smaller class of liquor-shops, were almost universally closed (on the last Sabbath), the beneficial effects of which are found in the comparative sparseness of our police reports."

All this has been done, and more will be, not by noisy vituperation, not by the impugning of motives, not by the aid of party, not by epithet or sarcasm, nor, indeed by the aid of new laws or special enactments, but by a quiet and calm, yet determined effort, to enforce already existing statutes ; at the same time, flooding the city with a plain statement of facts, with documentary evidence, and all, with that mildness of manner which those who are in the right can so well afford to exhibit, and hereby have given an example to the world of the power of firm and dignified action, as contrasted with bluster and bravado and noisy demagogueism, which last has so effectually retarded progress in other directions, and thrown back the wheels for half a century, while the actors themselves, in their rage, frothing, impotent, and atheistic, are a spectacle of pity to all men. And we heartily join in the utterance of a morning paper, that, "Great credit is due to the committee who have had the matin charge, for the discreet manner in which they have conducted their unyielding efforts to put an end to a demoralizing and wretched traffic. There has been neither fanaticism nor rancor in the proceedings of the committee; they have dealt with their opponents in a firm but Christian-like manner, and have prudently refrained from attempting to carry their scale of operations one inch beyond the point where they could be sustained not only by the law, but by the sanction of all prudent citizens, without regard to sect or party. If this Sunday liquor traffic can be abolished in this city, it will be through the means of such discreet and sagacious measures as have been taken to suppress it by the 'Sabbath Committee.'"

